# Nucleoside Metabolism Is Induced in Common Bean During Early Seedling Development

**DOI:** 10.3389/fpls.2021.651015

**Published:** 2021-03-25

**Authors:** Elena Delgado-García, Pedro Piedras, Guadalupe Gómez-Baena, Isabel M. García-Magdaleno, Manuel Pineda, Gregorio Gálvez-Valdivieso

**Affiliations:** ^1^Departamento de Botánica, Ecología y Fisiología Vegetal. Grupo de Fisiología Molecular y Biotecnología de Plantas. Campus de Excelencia Internacional en Agroalimentación, Campus de Rabanales, Edif. Severo Ochoa, Universidad de Córdoba, Córdoba, Spain; ^2^Departamento de Bioquímica y Biología Molecular, Campus de Excelencia Internacional en Agroalimentación, Campus de Rabanales, Edif. Severo Ochoa, Universidad de Córdoba, Córdoba, Spain; ^3^Servicio Central de Apoyo a la Investigación (SCAI), Unidad de Espectrometría de Masas y Cromatografía, Campus de Rabanales, Universidad de Córdoba, Córdoba, Spain

**Keywords:** nucleoside hydrolase, nucleoside degradation, *Phaseolus vulgaris*, germination, postgerminative development, nucleotide metabolism, ureides

## Abstract

Nucleoside hydrolases (NSH; nucleosidases) catalyze the cleavage of nucleosides into ribose and free nucleobases. These enzymes have been postulated as key elements controlling the ratio between nucleotide salvage and degradation. Moreover, they play a pivotal role in ureidic legumes by providing the substrate for the synthesis of ureides. Furthermore, nucleotide metabolism has a crucial role during germination and early seedling development, since the developing seedlings require high amount of nucleotide simultaneously to the mobilization of nutrient in cotyledons. In this study, we have cloned two nucleosidases genes from *Phaseolus vulgaris*, *PvNSH1* and *PvNSH2*, expressed them as recombinant proteins, and characterized their catalytic activities. Both enzymes showed a broad range of substrate affinity; however, PvNSH1 exhibited the highest activity with uridine, followed by xanthosine, whereas PvNSH2 hydrolyses preferentially xanthosine and shows low activity with uridine. The study of the regulation of nucleosidases during germination and early postgerminative development indicated that nucleosidases are induced in cotyledons and embryonic axes just after the radicle emergence, coincident with the induction of nucleases activity and the synthesis of ureides in the embryonic axes, with no remarkable differences in the level of expression of both nucleosidase genes. In addition, nucleosides and nucleobase levels were determined as well in cotyledons and embryonic axes. Our results suggest that PvNSH1 and PvNSH2 play an important role in the mobilization of nutrients during this crucial stage of plant development.

## Introduction

Nucleotides are molecules of crucial relevance in all living organisms. Besides being elementary components of RNA and DNA, they participate in bioenergetic processes, are part of cofactors for enzymes, and are components of secondary messengers and metabolites (Zrenner et al., 2006).

Purine and pyrimidine nucleotides can be synthesized *de novo* or through the salvage pathways (either from nucleosides and nucleobases released from nucleic acid and nucleotide metabolism), or been taken up from the soil, where they are found in significant amounts (Phillips et al., 1997; Jenkins et al., 2017). In terms of energy cost, the salvage pathways are cheaper than the *de novo* synthesis and may be important in non-growing cells that need to maintain their pools of nucleotides with little investment. In contrast, growing and dividing cells mainly depend on the *de novo* synthesis (Ashihara et al., 2020c). In plants, like in most animals and microorganisms, pyrimidine catabolism implies the reduction of uracil and thymine to CO_2_, NH_3_, and β-alanine or β-aminoisobutyrate, respectively (Ashihara et al., 2020b). On the other hand, plants can fully oxidize purine to glyoxylate, CO_2_, and NH_3_, in contrast to most animals, in which the main end products are urate or allantoin (Ashihara et al., 2020d). Purine nucleotide catabolism is particularly relevant in ureidic legumes; xanthine is the precursor of the ureides allantoin and allantoate, molecules that play an important role in the transport and storage of nitrogen in these legumes (Todd et al., 2006). Thus, when ureidic legumes are fixing nitrogen, ureides constitute almost the totality of the nitrogen transported from the nodules to the aerial plants (Díaz-Leal et al., 2012). Moreover, we have reported the importance of ureides during early seedling development in common bean (Quiles et al., 2009, 2019) and pointed to a connection between the catabolism of nucleic acids and nucleotides and ureides (Cabello-Diaz et al., 2012, 2015; Lambert et al., 2014, 2016). In addition, important roles of ureides in non-leguminous plants are been unveiled (Watanabe et al., 2014; Lescano et al., 2016; Soltabayeva et al., 2018; Takagi et al., 2018).

Throughout the past decade, the use of *Arabidopsis* mutants has allowed a considerable advance in our understanding on the pathways involved in nucleotide metabolism in plants (Witte and Herde, 2020). The salvage pathways and *de novo* synthesis meet at the formation of nucleoside monophosphates, the substrates of 5'-nucleotidases, phosphatases catalyzing the first step in the catabolic pathway. Purine nucleotide catabolism is mainly routed through GMP, which is dephosphorylated to guanosine and subsequently deaminated to xanthosine (Dahncke and Witte, 2013; Baccolini and Witte, 2019). In addition, direct dephosphorylation of xanthosine monophosphate (XMP) by an XMP phosphatase also contributes to the pool of xanthosine (Baccolini and Witte, 2019). Genes encoding GMP or XMP phosphatase have not been identified in *Arabidopsis* yet, but two phosphatases with high affinity for nucleosides monophosphate have been recently overexpressed and characterized in *Phaseolus vulgaris* (Cabello-Diaz et al., 2015; Galvez-Valdivieso et al., 2020), and an XMP phosphatase activity was identified in cowpea nodules (Atkins, 1981). The pyrimidine nucleotides UMP and CMP are dephosphorylated to the nucleosides uridine and cytidine, respectively, and the latter is deaminated to uridine (Zrenner et al., 2006).

Next step in nucleotide catabolism is catalyzed by nucleoside hydrolases (NSH), enzymes that cleave nucleosides into ribose and nucleobases. Nucleosidases have been postulated as key enzymes controlling the ratio between nucleotide salvage and degradation (Mohlmann et al., 2010). These enzymes have been purified and characterized from a number of plants, and enzymatic studies during some physiological stages have been performed (Ashihara et al., 2018). In plants, cytosolic and apoplastic nucleosidases have been found (Riewe et al., 2008; Jung et al., 2011). Cytosolic nucleosidases seem to be encoded by at least two genes (Riewe et al., 2008; Jung et al., 2009, 2011; Kopecna et al., 2013). In *Arabidopsis*, NSH1 is the only enzyme that hydrolyses uridine significantly (Riewe et al., 2008; Baccolini and Witte, 2019), and can also hydrolyze the purines xanthosine and inosine. Based on this, it was proposed that NSH1 is the main activity in purine and pyrimidine catabolism (Jung et al., 2011). However, the substrate specificity and the role of NSH2 was less clear (Jung et al., 2011; Riegler et al., 2011), except for the fact that it is induced under natural senescence (Jung et al., 2011). Recently, Baccolini and Witte (2019) demonstrated that NSH2 needs to interact with NSH1 to be active, and that xanthosine and inosine hydrolytic activities increase substantially when NSH1 activates NSH2 forming a heteromer. Furthermore, their work reveals that the NSH1-NSH2 heterocomplex is as effective as NSH1 degrading purine nucleosides, and the requirement of NSH2 for a correct regulation of purine catabolism.

Once the nucleobase and the ribose are cleaved, xanthine and hypoxanthine undergo several enzymatic reactions to bring out the ureides allantoin and allantoate, which can exert their roles or been totally catabolized (Munoz et al., 2011; Werner and Witte, 2011). On the other hand, uracil and thymine can also be degraded to recycle the nitrogen and the carbon skeleton (Zrenner et al., 2009).

Seed germination and postgermination stages are critical phases in plant life cycle. During these phases, seedlings develop from the reserves stored in the cotyledons, which are metabolized and transported to the developing axes. The knowledge of purine and pyrimidine metabolism during germination and postgerminative development is limited and based mainly on studies with radiolabeled precursors (Ashihara et al., 2018). These works showed that at the initial phase of germination, the salvage of nucleosides and nucleobases supports the synthesis of nucleotides and nucleic acids, whereas in the later stages, the demand of nucleic acid is mainly fulfilled by the *de novo* pathways, and the catabolic pathways increase to provide carbon and nitrogen to the growing seedling (Ashihara et al., 2020c). In addition, several studies demonstrated that nucleotide metabolism is strictly regulated at the early stages of seedling development to the point of being critical for the germination success (Stasolla et al., 2003; Jung et al., 2009; Cornelius et al., 2011). In common bean, nucleotidase activity and expression of two nucleotidase genes are induced during seedling development (Cabello-Diaz et al., 2012, 2015; Galvez-Valdivieso et al., 2020) coincidently with the induction of nucleases (Lambert et al., 2014, 2016).

We hypothesized that cytosolic nucleosidases play an important role in the mobilization of nutrients during the germination and early postgerminative development in the ureidic legume *P. vulgaris*. To evaluate this hypothesis, two cytosolic NSHs have been identified and cloned, both ORFs have been overexpressed, and the recombinant enzymes have been characterized. In addition, their regulation during germination and early postgerminative development in cotyledons and embryonic axes has been analyzed, as well as the level of nucleosides, nucleobases, ureides, and nuclease activity during these developmental stages. This work constitutes the first comprehensive analysis of nucleoside metabolism during germination and early seedling development in plants.

## Materials and Methods

### Biological Material and Growth Conditions

Plant tissues were isolated from common bean (*P. vulgaris* L.cv. Great Northern). Seeds were surface-sterilized and germinated as described by Galvez-Valdivieso et al. (2013), with the exception that seeds were kept in Petri dishes until the 5th day after the start of imbibition (DAI). Cotyledons, embryonic axes, and radicles were isolated at the indicated DAI, snap-frozen in liquid nitrogen, and stored at −80°C until used. For cotyledon senescence experiments, 3 DAI seedlings were transferred to pots containing perlite:vermiculite (2:1) and watered regularly with distilled water. For each experiment, at least three biological replicates were collected being each biological replicate a pool of tissues for at least three different seedlings.

*Nicotiana benthamiana* seeds were sown in a mix of peat moss (90%), lignofibre (5%), and cocopeat (5%), watered with tap water and grown in a controlled environment chamber at 26–21°C (light/dark) at 16 h light and 8 h dark photoperiod. Three to four-week-old plants were used for agroinfiltration.

### Identification and Cloning of Nucleosidase Genes in *Phaseolus vulgaris*

To identify *P. vulgaris* nucleosidase genes, *Arabidopsis thaliana* nucleosidase sequences were used to perform a Blast search in Phytozome database.^1^ This search allowed the identification of two sequences with high homology to *AtNSH1* and *AtNSH2*. Specific primers were designed and used to clone such cDNAs by Reverse Transcription PCR (RT-PCR; Supplementary Table S1). The PCR products were cloned in pSparkI (Canvax, Spain) and double strand sequenced.

### RNA Extraction and Real-Time Quantitative Reverse Transcription PCR

Total RNA extraction of plant tissues was performed using NZYol Reagent (NZYTech, Portugal) as indicated in Galvez-Valdivieso et al. (2013). RNA was treated with RNAse-free DNAseI (NEB, United States) and checked by PCR for the absence of contaminating genomic DNA. Single strand random-primed cDNA was synthesized using RevertAid Reverse Transcriptase (ThermoFisher Scientific, United States). Quantitative Reverse Transcription PCR (qRT-PCR) was performed as described previously (Diaz-Baena et al., 2020). Results were normalized to the geometric mean of *actin-2* and *ubiquitin* gene, and relative expression was calculated from 2^-∆∆CT^ values (Livak and Schmittgen, 2001). Primer specificity was verified by RT-PCR, sequencing the amplicons and following their dissociation curves. The sequence of the primers is shown in Supplementary Table S1.

### Cloning, Expression in *Escherichia coli*, and Purification of Recombinant Nucleosidases

The cDNAs containing the ORF of *PvNSH1* and *PvNSH2* were amplified by RT-PCR with the primers pET30-NSH1-SalI and pET30-NSH1-NotI, and pET30-NSH2-SalI and pET30-NSH2-NotI, respectively, both pairs inserting *Sal*I and *Not*I restriction sites (Supplementary Table S1). The PCR products were cloned into pSparkI (Canvax, Spain) and double strand sequenced. The inserts were released by digestion with *Sal*I and *Not*I, cloned in frame into pET30b+ (Novagen), and used to transform the *Escherichia coli* strain BL21 (DE3). Overexpression and purification of the recombinant proteins were performed as described in Galvez-Valdivieso et al. (2020) except that after the addition of IPTG, the culture was incubated at 28°C for 6 h, the binding buffer was 20 mM Na_2_HPO_4_, 500 mM NaCl, and 10 mM imidazole, (pH 7.4), supplemented with DNAase, RNAse, and 1 mM phenylmethylsulfonyl fluoride (PMSF) for the bacterial lysis, and the washing and the elution buffers contained 10 and 300 mM of imidazole, respectively.

### Immunization and Antibodies Preparation

Polyclonal antiPvNSH1 were generated by the Centralized Service of Experimental Animals (SAEX, Universidad de Córdoba, Spain). Two hundred micrograms of purified PvNSH1 protein were mixed with a volume of Freund’s complete adjuvant before injections in New Zealand white rabbits. Pre-immune serum was extracted before immunization for future controls. A second booster injection with 125 μg of protein emulsified in incomplete Freund’s adjuvant were applied 14 days after the first immunization and it was repeated monthly. The rabbits were bled for antiserum collection 10 days after each injection. Antibodies were enriched and concentrated by precipitation with ammonium sulphate and DEAE-Sephacel (GE Healthcare, United States) chromatography with 15 mM phosphate buffer pH 7.0. Antibodies were aliquoted and stored at −20°C.

### Transient Expression in *Nicotiana benthamiana* and Purification of the HAStrep-Tagged Proteins

The ORF of *PvNSH2* was amplified with pXCS-NSH2-HindIII and pXCS-NSH2-SmaI primers, which inserted *Hind*III and *Sma*I restriction sites, respectively. The PCR product was cloned into pSparkI (Canvax, Spain) and double strand sequenced. After digestion with *Hind*III and *Sma*I, the insert was cloned in frame into pXCS-HAStrep (Witte et al., 2004) and transformed into *Agrobacterium tumefaciens* GV3101::pMP90RK (Koncz and Schell, 1986). Fresh LB medium supplemented with the appropriate antibiotics was inoculated with an overnight culture, incubated at 28°C in a rotary shaker until it reached an OD_600nm_ of 0.4–0.6. At this point, the culture was harvested; cells were resuspended in infiltration buffer (10 mM MES pH 5.6; 10 mM MgCl_2_, 200 μM acetosyringone) to reach an OD_600nm_ of 0.4 and then incubated for 2 h at room temperature with gentle shaking. Identical procedure was followed with a culture of *A. tumefaciens* C58C1 + pCH32 carrying the pBIN61-P19 to express the P19 RNA silencing suppressor (Qu and Morris, 2002). Just prior inoculation, equal volumes of both cultures were mixed and infiltrated through the abaxial side of the leaves of 3 to 4-week-old *N. benthamiana* plants. Once infiltrated, the plants were maintained in the growth cabinet under the conditions described above until the infiltrated leaves were harvested, ground with liquid nitrogen, and stored at −80°C.

Ground leaf material was extracted with four volumes of extraction buffer [100 mM Tris-HCl (pH 8), 150 mM NaCl, 1 mM EDTA, 10 mM DTT, 0.5% Triton-X-100, 2% PVPP, and 1 mM PMSF], centrifuged at 20,000 *g* for 10 min at 4°C and at 100,000 *g* for 30 min at 4°C. The supernatant was transferred to a column with 200 μl of Strep Tactin sepharose suspension (GE Healthcare), washed with 10 volumes of washing buffer [50 mM Tris-HCl (pH 8), 150 mM NaCl, 1 mM EDTA, 1 mM DTT, 0.05% Triton-X-100, and 1 mM PMSF], and the recombinant PvNSH2 was eluted with elution buffer [10 mM Tris-HCl (pH 8), 150 mM NaCl, 1 mM DTT, 0.05% Triton-X-100, and 2.5 mM desthiobiotin].

### Protein Quantification

Protein concentration was determined using a BSA standard curve resolved on SDS-PAGE gel and stained using ProteoSilver Silver stain kit (Sigma-Aldrich). Band intensities were quantified with a Gel Doc EZ Imager (Biorad).

### Protein Gel Immunoblot Analysis

Protein gel immunoblot was performed as described in Galvez-Valdivieso et al. (2013). Briefly, proteins (15 μg) were resolved by 10% SDS-PAGE, transferred to a polyvinylidene fluoride (PVDF) membrane, and immunoblotted with the appropriate antibody. Alkaline phosphatase-conjugated anti-rabbit or anti-mouse IgG (Sigma) at a 1:12,000 dilution was used as secondary antibody. The blot was developed using NBT (4-nitroblue tetrazolium chloride; 250 mg ml^−1^) and BCIP (5-bromo-4-chloro-3-indolylphosphate; 125 mg ml^−1^) in 100 mM Tris-HCl (pH 9.5) and 5 mM MgCl_2_ buffer.

The primary antibodies were a 1:1,000 dilution of antiPvNSH1 produced in rabbit, a 1:3,000 dilution of monoclonal anti-polyhistidine antibody produced in mouse (Sigma-Aldrich), or a 1:5,000 dilution of monoclonal antibody against hemagglutinin (HA) produced in mouse (Sigma-Aldrich).

### Preparation of Crude Extracts

Plant tissues were ground with liquid nitrogen and extracts were obtained by adding four volumes of extraction buffer [50 mM Tris-HCl (pH 7.5), 0.15 mM sodium deoxycholate, 2 mM EDTA, 10% glycerol, 0.1% β-mercaptoethanol, and 0.5 mM PMSF]. After incubating 5 min at 4°C, the homogenate was clarified by centrifugation at 24,000 *g* for 10 min at 4°C, and the supernatant was considered as the crude extract.

### Determination of Nucleosidase Activity

Nucleosidase activity was determined by HPLC following the hydrolysis of nucleosides. Unless otherwise stated, the reaction mixture was performed in 50 mM Tris-HCl (pH 7.5), 1 mM DTT, and 2.5 mM substrate and initiated by the addition of an adequate amount of enzyme or crude extracts. The reaction was incubated at 40°C for 40 min. After that, the reaction was stopped by adding perchloric acid at a final concentration of 0.5 M and neutralized with KOH at the same final concentration. After removal of the precipitate by centrifugation at 16,000 *g*, 20 μl of the supernatant were injected in a Jasco HPLC system (Jasco, Tokyo, Japan) with a Jasco MD-2010 Plus Diode Array and a Chromolith Performance RP-18e (100–4.6 mm) column. Samples were resolved using an isocratic eluent with 50 mM ammonium acetate (pH 5.2) and 0.5% methanol (v/v) for uridine-uracil and cytidine-cytosine, 1% (v/v) for xanthosine-xanthine, inosine-hipoxanthine, and guanosine-guanine, and 2% (v/v) for thymidine-thymine. Nucleosides and nucleobases were detected at 254 nm.

### Protein Identification by Liquid Chromatography-Tandem Mass Spectrometry Analysis

In solution, protein digestion was performed as described in Gomez-Baena et al. (2019) using trypsin as protease. Peptides generated were analyzed by Liquid Chromatography-Tandem Mass Spectrometry Analysis (LC-MS/MS) in a nano LC system [Dionex Ultimate 3000 nano UPLC (Thermo Scientific)] attached to an Orbitrap Fusion mass spectrometer (Q-OT-qIT, Thermo Scientific). Tryptic peptides were loaded in an Acclaim Pepmap precolumn (300 μm × 5 mm, Thermo Scientific) for 5 min at 5 μl/min, using 2% acetonitrile/0.05% TFA as loading buffer. Peptides were then separated in a C18 Acclaim Pepmap column (75 μm × 50 mm, Thermo Scientific) at 40°C at 300 nl/min. Peptide separation was carried out over a 1 h gradient, using water and 0.1% formic acid as mobile phase A and 20% acetonitrile and 0.1% formic acid as mobile phase B. The gradient conditions were as follow: 0–120 min, from 4 to 35% B; 120–126 min, from 35 to 55% B; 126–129 min, from 55 to 90% B; 129–137 min, wash at 90% B; 137–152 min, and re-equilibration at 4% B. Eluting peptides were then analyzed in an Orbitrap Fusion (Thermo Scientific) operated in positive mode and set in top 40 data dependent acquisition (DDA) mode. The AGC ion count target was set to 2 × 10^3^ and the max injection time to 300 ms, Survey scans of precursors were obtained from 400 to 1,500 m/z at 120 K resolution (at 200 m/z) with a 4 × 10^5^ ion count target. Tandem MS was performed by quadrupole isolation at 1.2 Th. Only precursors with charge state 2–5 were sampled for MS^2^ setting a dynamic exclusion window of 15 s allowing a 10 ppm tolerance for selected precursor. Fragmentation was performed by CID at normalized collision energy of 35. Fragments were analyzed in the ion trap.

Raw data were processed using Peaks X (Bioinformatics Solutions). Peptides identification was performed selecting trypsin as protease for digestion, two missed cleavages allowed, carbamidomethylation of cysteines as fixed modification, and methionine oxidation as variable modification. For protein identification, we used a merged database containing all predicted proteins from *Nicotiana benthamiana* genome (downloaded from https://solgenomics.net/organism/Nicotiana_benthamiana/genome on October 30, 2020, containing 76,379 entries) and the NSH2 sequence from *P. vulgaris*. Precursor mass tolerance was set at 10 ppm and product ions tolerance at 0.5 Da. Peptide identifications were validated using a decoy database search and false discovery rate (FDR) was set at 1%.

### Metabolite Analyses by Liquid Chromatography-Mass Spectrometry Analysis

Metabolite analyses were carried out by means of an Acquity ultra performance liquid chromatograph (Waters, United States) coupled with a QTRAP 5500 triple quadrupole mass spectrometer (AB SCIEX, United States) and controlled with Analyst 1.6.2 software (AB SCIEX, United States). Nucleosides and nucleobases were separated by a Kinetex C18 column (150 × 2.10 mm, 5 μm, 100 Å) purchased from Phenomenex (Torrance, United States). Mobile phases were A: 0.01% (v/v) formic acid in water, and B: methanol. Fourteen target compounds were separated with the following gradient: 2% B for 2.0 min, a linear gradient to 50% B in 3.0 min, increase to 100% B in 5.0 min, 100% B for 2.0 min, back to the initial conditions in 0.1 min, and equilibrate for 5.0 min before next injections. The flow rate was 250 μl min^−1^, the injection volume 2 μl, and the column temperature 35°C.

The MS detection was operated in electrospray positive ionization mode and multiple reaction monitoring (MRM). The (M + H)^+^ precursor ions were used for the 14 compounds. The nebulizer and collision gas were nitrogen. The ion source parameters were identical for all the compounds: ion spray voltage operated at 5,500 V, the turbo gas temperature was 400°C, and the curtain gas (N2); ion source gas (GC1) and turbo gas (GS2) were set at 40, 50, and 50 psi, respectively. MRM ion transitions, final optimum declustering potential (DP), and collision energy (CE) providing the maximum signal intensity are summarized in Supplementary Table S3A.

Nucleotides and nucleosides were extracted by adding 5 ml of boiling water to 50 mg of frozen ground tissues. After boiling for 10 min, the mixture was incubated for 15 min in an ultrasonic bath and centrifuged at 250,000 *g* for 1 h at 4°C. The samples were diluted 20 times before measuring.

Standard stock solution of each analyte was prepared in water. Calibration standards were freshly prepared diluting the stock with mobile phase. The concentrations of calibration standards ranged from 0.6 to 12 ng/ml (five concentrations), except for adenosine and guanosine that ranged from 2.4 to 120 ng/ml (six concentrations). After preparation, these calibration standards were immediately injected using an autosampler refrigerated at 10°C. Linear regressions of 14 compounds showed correlation coefficient (*r*^2^) from 0.9950 to 0.9998. The limits of quantification (LOQ) for each analyte were determined at a signal-to-noise ratio (S/N) of about 10 (Supplementary Table S3B).

The accuracy of the developed method was determined using a recovery test. Known amounts of the 14 standards were added into a certain amount of ground *P. vulgaris* tissue. Then, three replicates of the spiked samples were extracted and analyzed as described above. To calculate the detected amounts (actual), the amount of each compound before spiking was subtracted from the total amount after spiking. The average recoveries were calculated as the ratio of detected amount (actual) to spiked amount (theoretical). The overall recoveries were between 86 and 103% with RSD less than 10%.

### Determination of Nuclease Activity

ssDNAse activity in the clarified extracts was assayed by measuring the release of acid soluble nucleotides from ssDNA as described Wood et al. (1998) with some modifications. The reaction mixture contained 50 mM TES (pH 5.5), BSA (1 mg/ml), and salmon testes DNA (0.5 mg/ml; Sigma-Aldrich) boiled for 10 min immediately prior to use, and an adequate amount of crude extract. The mixture was incubated at 35°C, and aliquots of 0.3 ml were extracted and mixed with 1 ml of 3.4% perchloric acid (v/v). After homogenization, the mixture was centrifuged at 24,000 *g* at 4°C for 15 min, the supernatant containing the released nucleotides was recovered, and its absorbance was measured at 260 nm. One unit of nuclease is defined as the amount of enzyme that produce a ∆OD_260_ = 0.01 min^−1^ (Yupsanis et al., 2004).

### Ureide Determination

Ureides were quantified using the colorimetric assay of glyoxylate derivatives (Vogels and Van der Drift, 1970), in which allantoin and allantoate are independently determined following their chemical conversion into glyoxylate. The sum of allantoin plus allantoate constitutes the total ureides value.

### Phylogenetic Analysis

The sequences were arranged with MUSCLE (Edgar, 2004), and the phylogenetic tree was built by MEGA X software (Kumar et al., 2018), based on Neighbor-Joining method with a bootstrap value of 1,000 replicates. NSH sequences were obtained from Phytozome, the *Arabidopsis* Information Resource (TAIR), and the National Center for Biotechnology Information databases (NCBI). The aligned sequence were: *E. coli* Ybek (AAN79208); *E. coli* Yeik (AAA60514); *Crithidia fasciculata* (CFU43371); *Saccharomyces cerevisiae* (AF217406); *Synechococcus* (CP000239); *Trypanosoma vivax* (AF311701); *Volvox carteri* (Vocar0002s0393); *A. thaliana* AtNSH1 (At2g36310) and AtNSH2 (At1g05620); *Cicer arietinum* NSH1 (Ca_00782) and NSH2 (Ca_19997); *Chlamydomonas reinhardtii* (Cre06g271050); *Glycine max* NSH1.1 (XM014773909), NSH1.2 (Glyma.19G193700), NSH2.1 (Glyma.20g000200), and NSH2.2 (Glyma.09 g285900); *Lotus japonicus* NSH1 (Lj1g0000699.1) and NSH2 (Lj2g0019610.1); *Medicago truncatula* NSH1 (Medtr07g104270), NSH2.1 (Medtr1g039400), and NSH2.2 (Medtr1g039410); *Nicotiana tabacum* NSH1 (XM_016656799) and NSH2 (XM_016637716); *Oryza sativa* NSH1.1 (Os08g44370), NSH1.2 (Os09g39440), and NSH2 (Os03g31170); *P. vulgaris* PvNSH1 (MW435747) and PvNSH2 (MW435748); *Physcomitrella patens* PpNRH1 (JQ649322), PpNRH2 (JX861385), and PpNRH3 (JX861386); *Populus trichocarpa* NSH1 (Potri.006g083400) and NSH2 (Potri.007g144600); *Solanum lycopersicum* NSH1 (Solyc09g009690) and NSH2 (Solyc12g018990); *Triticum aestivum* NSH1.1 (Traes_7DS_DFF4A032D.1), NSH1.2 (Traes_1AS_FDC3AF8A2.1), and NSH2 (Traes_3AS_E563BAA68.1); *Vigna unguiculata* NSH1 (Vigun01g172400) and NSH2 (Vigun02g010300); *Zea mays* ZmNRH1a (HQ825159), ZmNRH1b (HQ825160), ZmNRH2a (HQ825161), ZmNRH2b (JQ594984), and ZmNRH3 (HQ825162).

Phylogenetic tree was edited with iTOLv4 (Letunic and Bork, 2019) and sequence logos were generated with Skylign (Wheeler et al., 2014).

## Results

### Isolation and Characterization of *Phaseolus vulgaris* Nucleosidase Genes

A Blast search using *Arabidopsis thaliana* NSH1 nucleosidase on Phytozome database identified two genes that encode putative NSH in *P. vulgaris* (*Phvul.001G188700* and *Phvul.003G000600*). Specific primers were designed and the cDNAs encoding these genes were cloned and sequenced. Because of their homologies with *Arabidopsis AtNSH1* and *2* nucleosidases, the *Phaseolus* genes were named *PvNSH1* and *PvNSH2*.

**Figure 1:**
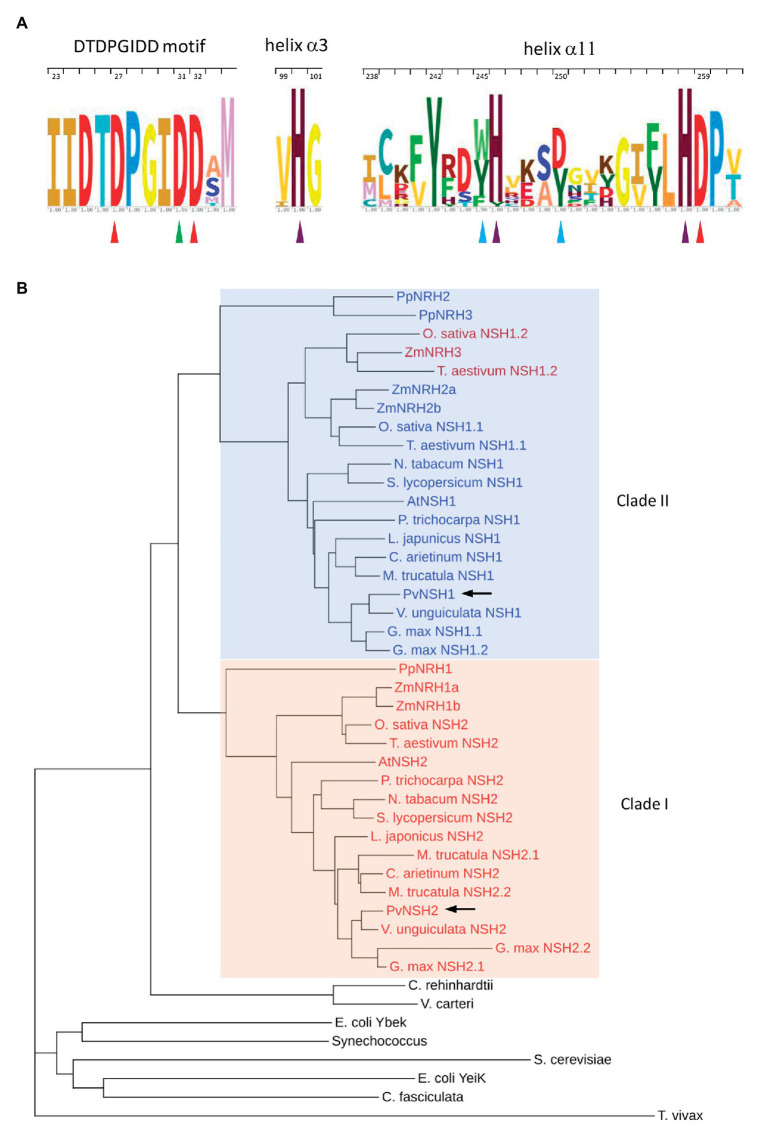
Conservation of the amino acid sequences of nucleoside hydrolases (NSHs) from plants and cladogram. **(A)** Sequence logos of the DTDPGIDD motif and the α3 and α11 helix. Red triangles indicate the Asp residues involved in calcium ion coordination. The Asp-binding 2-OH group of ribose is highlighted with a green triangle. Turquoise triangles indicate the crucial residues involved in substrate binding in plants NSHs. Purple triangles highlight the conserve histidines. Residues are numbered according to PvNSH1 sequence. **(B)** Cladogram highlighting the two distinct clades. The color of the text indicate the subclass [blue: subclass II (Asp-250 + Trp-245); red: subclass Ia (Tyr-250 + Tyr-245), and dark red: subclass Ib (Asp-250 + Trp-245), as proposed in Kopecna et al., 2013]. *Phaseolus vulgaris* nucleosidases are highlighted in with arrows.

PvNSH1 and PvNSH2 exhibited a similarity of 52.6% and contain 334 and 323 amino acids with an estimated molecular mass of 35.7 and 34.6 kDa, respectively. As all plant nucleosidases, both proteins conserved the N-terminal aspartate cluster (DTDPGIDD; Versees and Steyaert, 2003). In addition, PvNSH1 and two conserve other amino acids identified as important for the nucleosidase activity in the crystal structures of *Physcomitrella patens* PpNRH1 and *Zea mays* ZmNRH3 (Figure 1A). Phylogenetic analysis showed that plant nucleosidases cluster in two clades (Figure 1B). PvNSH1 is in clade II, comprising mainly subclass II, which groups nucleosidases carrying Asp at position 250 and Trp at 245 (PvNSH1 notation), which is associated with uridine and xanthosine preference. On the contrary, PvNSH2 belongs to Clade I, formed by nucleosidases from the subclass Ia, containing Tyr-250 and Tyr-245 (PvNSH1 notation) and having xanthosine and inosine preference (Kopecna et al., 2013). *Zea mays* ZmNRH3, *T. aestivum* NSH1.2, and *O. sativa* NSH1.2 are in clade II but belong functionally to subclass Ib (Tyr-250 and Trp-245) and prefer xanthosine and inosine as substrates. PvNSH1 and PvNSH2 share 90–70% sequence similarity with plant NSHs grouped in their same clade and around 50% with the proteins in the other branch (Supplementary Table S2).

### Common Bean NSH Overexpression and Characterization

**Figure 2:**
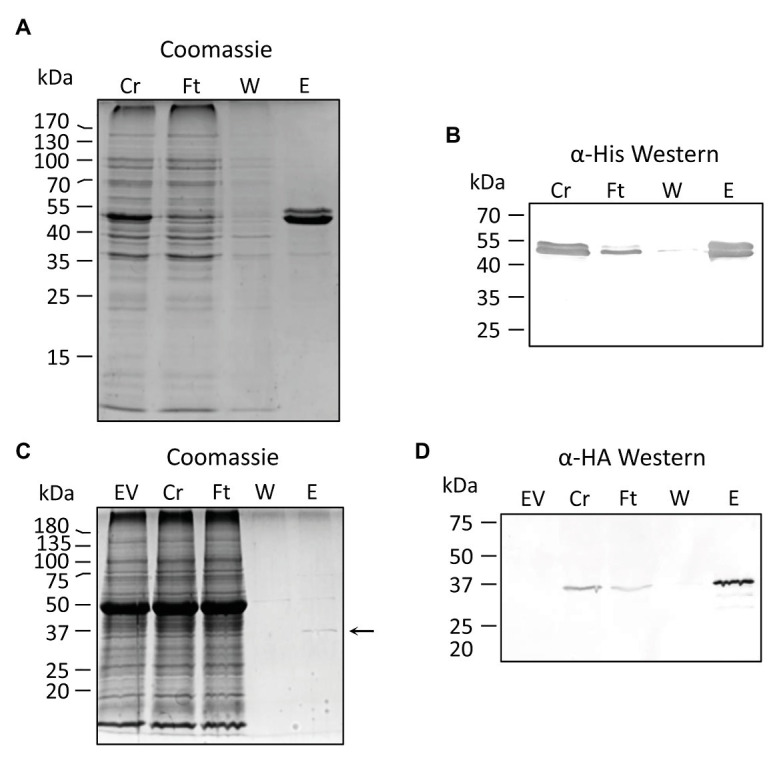
Purification of recombinant PvNSH1 and PvNSH2. **(A,B)** Purification of His-tagged PvNSH1 overexpressed in *Escherichia coli* **(A)** Colloidal Coomassie stained and **(B)** Western blot. **(C,D)** Purification of Strep-tagged PvNSH2 overexpressed in *Nicotiana benthamiana* leaves **(C)** Colloidal Coomassie stained and **(D)** Western blot. The arrow points at the protein band representing PvNSH2. Cr, Crude extract; Ft, flowthrough; W, wash; E, elution, and EV, empty vector.

PVNSH1 was purified after its overexpression as recombinant protein in *E. coli* (Figures 2A,B). The purified protein showed nucleosidase activity and it was used to perform substrate specificity assays. PvNSH1 was more active toward uridine and xanthosine (53.4% of the activity with uridine), whereas it exhibited weaker activity with inosine (21.4% of the activity with uridine; Table 1). Residual activity was observed with guanosine (3.2%), whereas no activity was detected when cytidine and thymidine were used as substrates. No activity with adenosine could be assayed given the lack of optimal resolution of the HPLC column.

**Table 1:** Substrate specificity of PvNSH1 and PvNSH2.

Substrate	Relative activity	
	PvNSH1	PvNSH2
Xanthosine	53.4 ± 4.0	100 ± 11.0
Inosine	21.4 ± 3.0	18.9 ± 0.3
Guanosine	3.2 ± 0.0	4.5 ± 0.0
Uridine	100 ± 9.7	13.1 ± 2.0
Citydine	ND	ND
Thymidine	ND	ND

Enzymatic activity was measured at a 2 mM substrate concentration. Activity with uridine for PvNSH1 (specific activity 44.4 U mg^−1^) and xanthosine for PvNSH2 (specific activity 23.0 U mg^−1^) was taken as 100%.

The same strategy was followed to purify PvNSH2 overexpressed in *E. coli*. Nevertheless, most of the protein precipitated with the insoluble fraction and we were unable to determine nucleosidase activity with the low amount of PvNSH2 found in the soluble fraction (Supplementary Figure S1). To circumvent this problem, PvNSH2 was transiently overexpressed in *N. benthamiana*, and the recombinant protein was purified (Figures 2C,D). Purified PvNSH2 showed nucleosidase activity and the substrate specificity of the purified enzyme showed the highest activity with xanthosine, whereas, the activity with uridine and inosine was much weaker (Table 1). The activity with guanosine was very low and no activity was detected with cytidine or thymidine as substrates. Therefore, the purified PvNSH2 showed nucleosidase activity with several nucleosides, contrasting to the *Arabidopsis* NSH2, which needs to interact with NSH1 to be active (Riegler et al., 2011; Baccolini and Witte, 2019). A possible explanation could be that the transiently expressed PvNSH2 interacted with the native *N. benthamiana* NSH1 forming an active heteromer and co-purified together. To explore this possibility, the fraction containing the purified PvNSH2 was analyzed by LC–MS/MS. PvNSH2 was undoubtedly identified covering 72% of the protein sequence with 29 unique peptides identified (Supplementary Table S4). However, no confident identification for NSH1 was obtained indicating that *N. benthamiana* NSH1 either is not co-purifying with PvNSH2 or its amount is under the limit of detection of the technique, and far lower than the amount of purified PvNSH2. We also tested if PvNSH1 could interact with PvNSH2 by incubating extracts of *E. coli* overexpressing PvNSH1-His with extracts of *N. benthamiana* overexpressing PvNSH2-HAStrep and checking if the PvNSH1-His could be co-purified by affinity purification of the Strep-tagged PvNSH2. However, no immunoreaction was observed with anti-His antibody after purification (Supplementary Figure S2). All these results strongly suggest that PvNSH2 does not need to interact to PvNSH1 to be active, although we cannot fully discard that PvNSH2 could also form heteromers with PvNSH1.

Then, we addressed the characterization of the kinetic properties of PvNSH1 and PvNSH2. All the substrates assayed showed a regular Michaelis-Menten behavior. For PvNSH1, the apparent *K*_m_ values for xanthosine, uridine, and inosine hydrolysis were 0.64, 2.02, and 0.99 mM, with a higher catalytic constant (k_cat_) for uridine and lower for inosine but with relatively similar catalytic efficiency for all the substrates (Table 2; Supplementary Figures S3A,C,E,G).

**Table 2:** Kinetics parameters for PvNSH1 and PvNSH2.

	PvNSH1			PvNSH2		
Substrate	K_m_ (mM)	k_cat_ (s^−1^)	k_cat_/K_m_ (s^−1^·mM^−1^)	K_m_ (mM)	k_cat_ (s^−1^)	k_cat_/K_m_ (s^−1^·mM^−1^)
Xanthosine	0.64 ± 0.11	20.41 ± 1.42	32.04 ± 3.49	0.12 ± 0.02	13.18 ± 0.26	116.01 ± 15.19
Inosine	0.99 ± 0.04	10.35 ± 0.40	10.48 ± 0.01	0.21 ± 0.02	2.91 ± 0.14	13.67 ± 0.64
Uridine	2.02 ± 0.27	62.88 ± 7.60	31.21 ± 0.44	–	–	–

The apparent K_m_ of PvNSH2 for xanthosine and inosine was 0.12 and 0.21 mM, respectively, which is around five times lower than that of PvNSH1 (Table 2; Supplementary Figures 3B,D,F). The catalytic efficiency (k_cat_/K_m_) of PvNSH2 for xanthosine was more than three times higher than the catalytic efficiency of PvNSH1.

### Regulation of *Phaseolus vulgaris* Nucleosidases During Germination and Early Postgerminative Development

**Figure 3:**
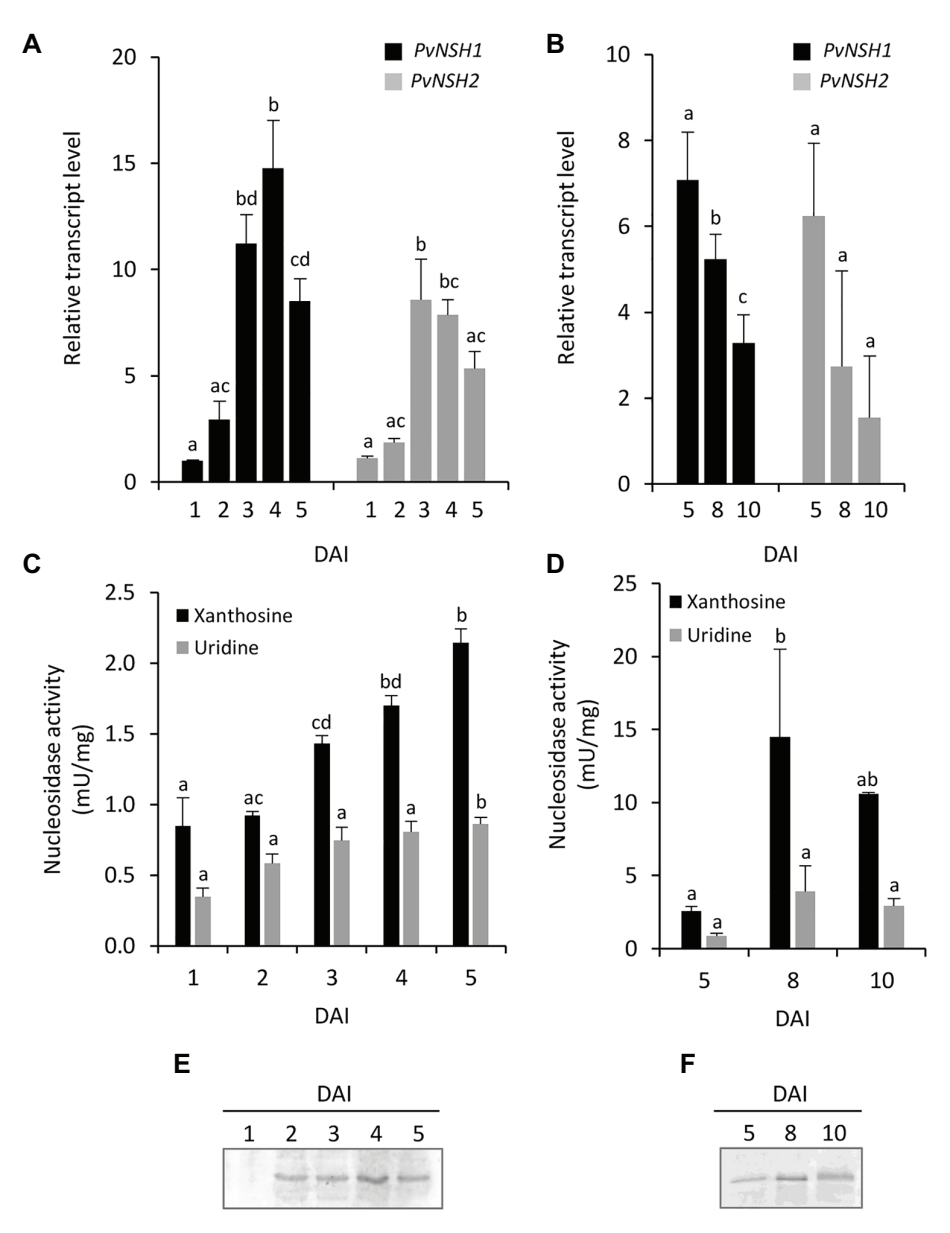
Nucleosidase regulation during germinative and early postgerminative development of cotyledons. **(A,B)** Evolution of *PvNSH1* and *PvNSH2* mRNA levels, **(C,D)** nucleosidase activity, and **(E,F)** immunodetection of nucleosidase during germination and early postgerminative development. Real-time Quantitative Reverse Transcription PCR (qRT-PCR) results were normalized with the geometric mean of *actin-2* and *ubiquitin* genes and expressed relative to the expression of *PvNSH1* in cotyledons at 1 DAI, which was considered the value 1. Nucleosidase activity was assayed as described in the section “Materials and Methods” using xanthosine or uridine as substrate. Nucleosidase immunodetection was performed with polyclonal anti-PvNSH1 antibodies. Equal amounts of total protein (15 μg) were loaded per lane in all the gels. Values are mean ± SE of at least three biological replicates. Different letters indicate significant differences among the developmental stages for the same gene or substrate as analyzed by ANOVA followed by Tukey’s *post hoc* analysis (*p* ≤ 0.05).

To analyze the regulation of common bean nucleosidases in cotyledons during germination, early seedling development and senescence, the expression level of *PvNSH1* and *PvNSH2*, nucleosidase activity with xanthosine, and the abundance of PVNSH1 and 2 were analyzed. *PvNSH1* and *PvNSH2* were detected 1 DAI, and their expression boosted on the third DAI, coincident with the radicle emergency (Figures 3A,B), with no remarkable differences between both genes. Once the radicle was visible, the amount of transcript of both genes decreased. Nucleosidase activity in cotyledons was detected in all the analyzed samples and increased after the radicle emergency (3 DAI) but, on the contrary to transcript accumulation, NSH specific activity continued increasing to reach a maximal activity at 8 DAI and stayed still high at 10 DAI, when the cotyledons are already shrunk (Figures 3C,D). Western blot analysis revealed accumulation of protein at 2 DAI, just before the radicle emerged, and even higher accumulation at 4 DAI (Figure 3E), remaining still high during cotyledon senescence (Figure 3F).

**Figure 4:**
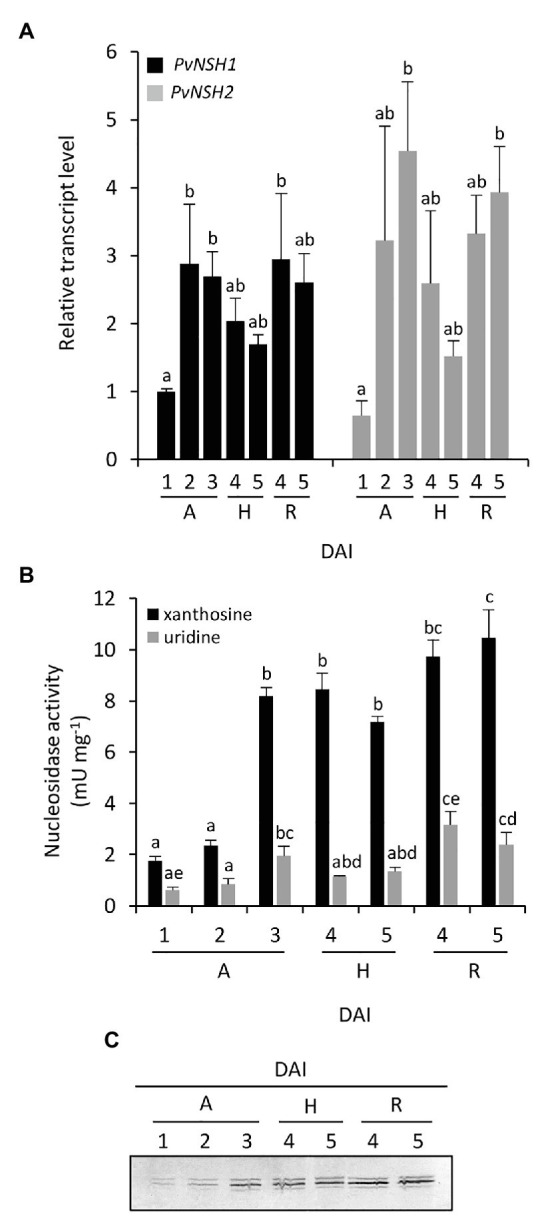
Nucleosidase regulation during germinative and early postgerminative development of embryonic axes, radicles and hypocotyls. **(A)** Evolution of *PvNSH1* and *PvNSH2* mRNA levels, **(B)** nucleosidase activity, and **(C)** immunodetection of nucleosidase during germination and early postgerminative development. Real-time qRT-PCR results were normalized with the geometric mean of *actin-2* and *ubiquitin* genes and expressed relative to the expression of *PvNSH1* in axes at 1 DAI, which was considered the value 1. Nucleosidase activity was assayed as described in the section “Materials and Methods” using xanthosine or uridine as substrate. Nucleosidase immunodetection was performed with polyclonal anti-PvNSH1 antibodies. Equal amounts of total protein (15 μg) were loaded per lane in all the gels. Values are mean ± SE of at least three biological replicates. Different letters indicate significant differences among the developmental stages gene or substrate as analyzed by ANOVA followed by Tukey’s *post hoc* analysis (*p* ≤ 0.05). A, embryonic axes; H, hypocotyls; and R, radicles.

The regulation of common bean nucleosidases was also analyzed in embryonic axes during germination and early seedling development. Radicle and hypocotyl were distinguishable at 4 DAI, so we tested the regulation of nucleosidases in complete embryonic axes from 1 to 3 DAI and in isolated hypocotyls and radicles from 4 to 5 DAI. In embryonic axes, the expression pattern of *PvNSH1* and *2* followed a trend similar to that found in cotyledons, with both genes reaching a maximum between 2 and 3 DAI (Figure 4A). Nucleosidase specific activity in embryonic axes was higher than in cotyledons, and both, nucleosidase activity and protein accumulation also increased after radicle emergency (Figures 4B,C). It is noticeable that in addition to the strongest signal, the immunoreaction revealed two weaker bands just below and above the main signal, although, given than the antiPvNSH1 antibodies showed cross reaction with PvNSH2, we cannot discern if they correspond to different nucleosidases or posttranslational modification. At 4 DAI hypocotyls and radicles, transcript accumulation and nucleosidase activity were similar to that of 3 DAI embryonic axes, whereas at 5 DAI, the mRNA level and nucleosidase activity seem to decrease in hypocotyls (Figures 4A,B).

When nucleosidase activity was assayed using uridine as substrate, the trend was like the one observed with xanthosine, but the specific activity was considerably lower, with this difference being more marked in embryonic axes than in cotyledons (Figures 3, 4).

### Nuclease Activity During Germination and Early Postgerminative Development

**Figure 5:**
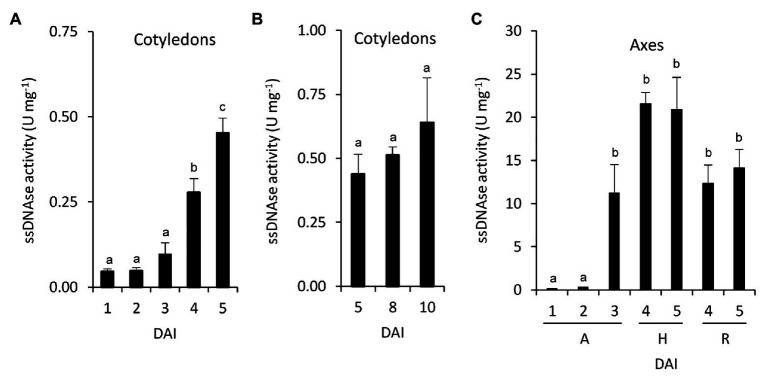
Nucleic acid degradation during germinative and early postgerminative development in common bean. ssDNA activity was determined in cotyledons **(A,B)**, and embryonic axes **(C)**. Values are mean ± SE of at least three biological replicates, with two technical replicates for experiment. Different letters indicate significant differences among the developmental stages as analyzed by ANOVA followed by Tukey’s *post hoc* analysis (*p* ≤ 0.05). DAI, days after the start of the imbibition; A, embryonic axes; H, hypocotyls; and R, radicles.

To explore the relation between the induction of nucleosidases and nucleic acid degradation, total *in vitro* nuclease activity from common bean seedlings was determined. Cotyledons ssDNAse activity increased after the radicle emergency and did not change significantly during nutrient mobilization and cotyledon senescence phases (Figures 5A,B). In embryonic axes, ssDNAse also increased after the radicle emerged (Figure 5C). At 4 and 5 DAI, the activity in hypocotyls tend to be higher than in radicles.

### Accumulation of Ureides During Germination and Early Postgerminative Development

**Figure 6:**
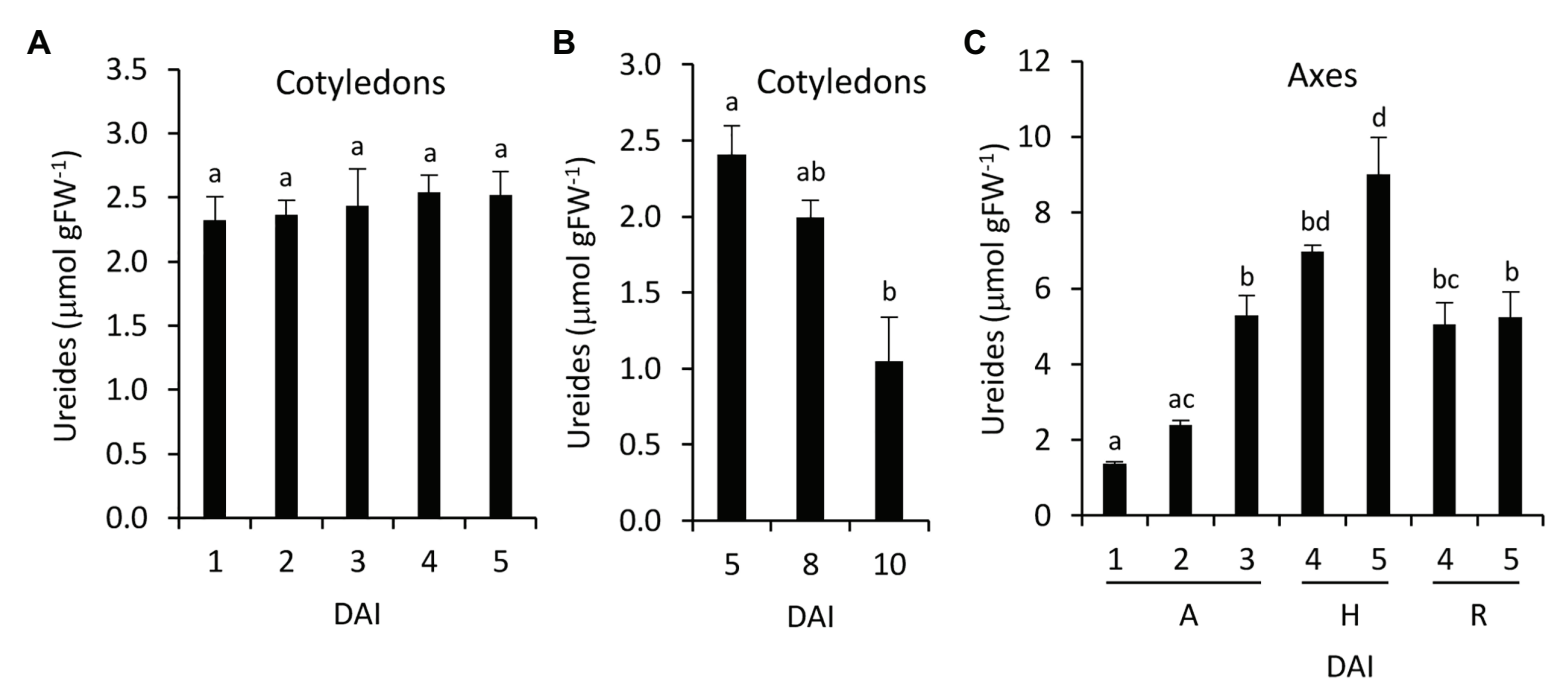
Ureides accumulation during germinative and early postgerminative development in common bean. Ureides accumulation was determined in cotyledons **(A,B)** and embryonic axes **(C)**. Values are mean ± SE of at least three biological replicates. Different letters indicate significant differences among the developmental stages as analyzed by ANOVA followed by Tukey’s *post hoc* analysis (*p* ≤ 0.05). DAI, days after the start of the imbibition; A, embryonic axes; H, hypocotyls; and R, radicles.

Total ureides concentration was determined in cotyledons and embryonic axes during germination and seedling development. In cotyledons, ureides did not change during germination but decrease when the cotyledons senescence (Figures 6A,B), whereas ureides increase significantly in embryonic axes between 2 and 3 DAI (Figure 6C). This increase continues in hypocotyls whereas in radicles remain lower.

### Nucleosides and Nucleobases Content During Germination and Early Postgerminative Development

To investigate the distribution of nucleosides and nucleobases during germination and postgerminative development, these were quantified in cotyledons and embryonic axes using HPLC-MS. In cotyledons, adenosine was the most abundant nucleoside, followed by guanosine, with none of them changing remarkably from 1 to 5 DAI (Table 3A). Of the better substrates of nucleosidases, uridine was the most abundant, and its concentration decreased at 2 DAI, whereas xanthosine was only detectable at 1 DAI and inosine was below the limit of quantification. Adenine, uracil, and xanthine were the only nucleobases detected, being xanthine the most abundant. Adenine levels were reduced at 5 DAI, uracil was only detected at 1 DAI, whereas xanthine was undetectable from the 2 DAI. Adenine and uracil levels were significantly lower than the corresponding nucleosides, whereas xanthine was in the same order than xanthosine. In embryonic axes, adenosine was also the most abundant nucleoside, followed by guanosine and uridine (Table 3B). These three nucleosides showed a tendency to increase from 1 to 3 DAI, being this trend more marked in adenosine. When radicle and hypocotyl differentiated, the level of nucleosides remained stable in hypocotyls, whereas they decreased in radicles. Xanthosine was only detectable in 1 DAI embryonic axes. Adenine and xanthine were detected in embryonic axes with similar levels and distribution as in cotyledons.

**Table 3:** Nucleosides and nucleobases content in common bean seedlings.

a) Cotyledons					
	Metabolite content (μg gFW^−1^)				
	Cotyledons				
	1 DDI	2 DDI	3 DDI	4 DDI	5 DDI
Xanthosine	7.2 ± 2.0	–	–	–	–
Inosine	–	–	–	–	–
Uridine	11.8 ± 0.5^a^	5.7 ± 0.1^b^	8.1 ± 0.7^b^	5.4 ± 0.56^b^	5.9 ± 0.6^b^
Adenosine	91.0 ± 6.3^a^	78.7 ± 3.0^a^	80.2 ± 3.9^a^	75.3 ± 5.8^a^	77.4 ± 4.1^a^
Guanosine	18.0 ± 1.1^a^	21.0 ± 1.3^a^	22.8 ± 1.5^a^	21.3 ± 1.7^a^	22.5 ± 1.5^a^
Thymidine	–	–	–	–	–
Cytidine	0.8 ± 0.1^a^	1.1 ± 0.1^ab^	1.5 ± 0.2^b^	0.9 ± 0.1^a^	1.2 ± 0.1^ab^
Xanthine	10.2 ± 0.9^a^	11.5 ± 3.5^a^	–	–	–
Hypoxanthine	–	–	–	–	–
Uracil	1.8 ± 0.4	–	–	–	–
Adenine	1.9 ± 0.0^ac^	2.2 ± 0.1^ab^	2.9 ± 0.1^b^	1.8 ± 0.3^ac^	1.4 ± 0.1^c^
Guanine	–	–	–	–	–
Thymine	–	–	–	–	–
Cytosine	–	–	–	–	–

b) Embryonic axes, hypocotyls, and radicles							
	Metabolite content (μg gFW^−1^)						
	Axes			Hypocotyls		Radicles	
	1 DDI	2 DDI	3 DDI	4 DDI	5 DDI	4 DDI	5 DDI
Xanthosine	5.8 ± 1.0	–	–	–	–	–	–
Inosine	–	–	–	–	–	–	–
Uridine	5.2 ± 0.6^a^	9.4 ± 0.3^a^	23.5 ± 1.4^b^	23.45 ± 1.7^b^	24.7 ± 1.8^b^	11.2 ± 0.3^a^	12.3 ± 2.9^a^
Adenosine	42.4 ± 6.1^a^	76.1 ± 3.2^ab^	163.3 ± 22.7^b^	155.6 ± 22.9^b^	159.4 ± 27.2^b^	78.5 ± 20.6^ab^	81.1 ± 32.2^ab^
Guanosine	42.9 ± 6.7^a^	50.0 ± 1.4^a^	139.4 ± 6.5^b^	129.2 ± 12.8^b^	127.8 ± 18.6^b^	64.9 ± 8.5^a^	56.37 ± 17.7^a^
Thymidine	–	–	–	–	–	–	–
Cytidine	2.1 ± 0.1^a^	2.6 ± 0.3^ab^	7.9 ± 1.3^ab^	9.4 ± 1.2^ab^	10.1 ± 2.6^b^	4.5 ± 1.0^ab^	6.5 ± 2.4^ab^
Xanthine	8.0 ± 1.8	9.3 ± 2.2	–	–	–	–	–
Hypoxanthine	–	–	–	–	–	–	–
Uracil	–	–	–	–	–	–	–
Adenine	1.3 ± 0.2^a^	3.7 ± 0.2^b^	3.1 ± 0.3^ab^	2.1 ± 0.1^ab^	1.9 ± 0.0^ab^	1.5 ± 0.0^a^	3.2 ± 1.1^ab^
Guanine	–	–	0.6 ± 0.0^a^	0.8 ± 0.1^a^	1.4 ± 0.6^a^	–	0.9 ± 0.3^a^
Thymine	–	–	–	–	–	–	–
Cytosine	–	–	–	–	–	–	–

Metabolites were determined in extracts from tissues of the indicated days after the start of the imbibition (DAI) of common bean seedlings. Data show mean ± SD (3 ≤ *n* ≤ 12). Different letters indicate significant differences among the developmental stages as analyzed by ANOVA followed by Tukey’s *post hoc* analysis (*p* ≤ 0.05). – indicates metabolites below the quantification limit.

## Discussion

The *de novo* synthesis, salvage and degradation of nucleotides are processes that need to be highly coordinated for the successful development of plants. Thus, misregulation of genes involved in these processes causes abnormalities in plant development as dwarfism, altered fertility, zygote lethality, reduced germination, and altered photosynthetic rate among others (Witte and Herde, 2020).

Nucleoside hydrolases, enzymes that catalyze the hydrolysis of nucleosides into ribose and nucleobase, have been postulated as key enzymes controlling the balance between nucleotide salvage and catabolism (Mohlmann et al., 2010). Analysis of the genome databases showed that nucleosidases are encoded by two genes in most plant species, although some of them as soybean, rice, wheat, and maize contain three or more copies. Maize and rice cases can be explained because they evolved from an ancestor that suffered a whole genome duplication (Gaut and Doebley, 1997; Yu et al., 2005), whereas the genomes of soybean and wheat were recently duplicated (Schmutz et al., 2010; Brenchley et al., 2012). The genome of *P. vulgaris* encodes two cytosolic nucleosidases, PvNSH1 and 2, that shares a 53% of sequence similarity, which is lower than the presented with their respective orthologous in other plant species, suggesting that the two paralogues appeared early in the evolution. Both conserve the aspartate cluster DXDXXXDD presents in all nucleosidases characterized until now (DTDPGIDD in plants; Versees and Steyaert, 2003), and all the residues identified as important for the nucleosidase activity, including the three Asp and the Leu that coordinate with the Ca^2+^ cofactor, the residues that bind the ribose moiety, and the amino acids that surround the nucleobase (Figure 1A). From all these conserved residues, there are only two that differ between PvNSH1 and 2; PvNSH1 Trp-245 and Asp-250 are substituted by Tyr-234 and Tyr-239 in PvNSH2. These two residues have an essential role determining the specificity of substrate (Kopecna et al., 2013). Phylogenetic analysis shows that common bean nucleosidases cluster in two different clades (Figure 1B), thus confirming the correlation between the clustering and the enzymatic properties reported by Kopecna et al. (2013). Thus, Clade I comprise the Subclass Ia, that includes nucleosidases with inosine and xanthosine preference (Tyr-250 and Tyr-245), and Clade II comprise mainly the Subclass II (Asp-250 and Trp-245) with nucleosidases preferring uridine-xanthosine. This agrees with the substrate specificity experimentally observed in common bean, in which PvNSH1 presents a much higher activity for uridine than PvNSH2, and with the reported for other recombinant nucleosidases from plants (Jung et al., 2009, 2011; Kopecna et al., 2013).

Purified recombinant PvNSH1 and PvNSH2 can hydrolyze both, purine and pyrimidine nucleosides but cannot hydrolyze all nucleosides (Table 1). PvNSH1 shows the highest specific activity with uridine, followed by xanthosine and, in a lower degree, inosine (Table 2). This concurs with the substrate specificity of other nucleosidases included in Subclass II, as *Arabidopsis* NSH1, maize ZmNRH2a and b, and *Physcomitrella* PpNSH2 (Jung et al., 2009, 2011; Kopecna et al., 2013; Baccolini and Witte, 2019). PvNSH1 specificity constant (k_cat_/K_m_) for the three nucleosides is in the same order, which suggests that this enzyme has similar preference for all these substrates. By contrast, PvNSH2 presents higher hydrolytic activity with xanthosine and lower with inosine and uridine, with an order of magnitude higher specificity constant for xanthosine than for inosine, suggesting that this enzyme has a clear preference for xanthosine as substrate. When compared to PvNSH1, it is noticeable that PvNSH2 specificity constant for xanthosine was more than three times higher than that of PvNSH1 and its K_m_ around five times lower.

Moreover, both common bean NSHs showed residual activity with guanosine and no activity with cytidine and thymidine, supporting previous studies that indicate that guanosine and cytidine degradation needs their deamination to xanthosine and uracil, respectively, before been hydrolyzed (Moffatt et al., 2002; Stasolla et al., 2003; Dahncke and Witte, 2013). Conversion of thymidine to thymine has been detected in ^14^C-thymidine feeding studies (Katahira and Ashihara, 2002), but no *in vitro* hydrolase activity has been reported to date.

When PvNSH2 was overexpressed in *E. coli*, most of the protein remains in the insoluble fraction (Supplementary Figure S1), and we were unable to detect nucleosidase activity in the small amount of soluble fraction. Formation of inclusion bodies after overexpression in *E. coli* was previously described for *Arabidopsis* NSH2 (Riegler et al., 2011), and none or residual activity could be assayed in the soluble fraction (Jung et al., 2011; Riegler et al., 2011). However, its overexpression in *N. benthamiana* allowed the purification of soluble AtNSH2 (Baccolini and Witte, 2019) but, in contrast to what we observed for PvNSH2, the *Arabidopsis* enzyme does not show hydrolytic activity with any of the substrates assayed, and needs to form a complex with AtNSH1 to be active. Analysis of the elution fractions containing the purified PvNSH2 by LC-MS/MS did not show evidence of the co-elution of *N. benthamiana* NSH1 (Supplementary Table S4). Besides, we were unable to detect PvNSH1-PvNSH2 interaction by co-purifying extracts overexpressing both proteins (Supplementary Figure S2). Taken together, our results strongly suggest that the possibility of PvNSH2 interacting with *N. benthamiana* NSH1 to form an active heteromer and co-purifying together is very low, and that PvNSH2 does not need to interact with PvNSH1 to be active. Interestingly, PpNH1, a nucleoside hydrolase from the moss *P. patens* with high xanthosine and low uridine activity forms homodimers (Kopecna et al., 2013). One can speculate that this difference between common bean and *Arabidopsis* NSH2 may be due to the special role of purine nucleotides in ureidic legumes (Todd et al., 2006; Quiles et al., 2019) that may have driven to the specialization of this isoform. Nevertheless, we cannot exclude the possibility that under certain conditions PvNSH2 could also form heteromers with PvNSH1.

Seed germination is a physiological stage characterized by marked fluctuations in nucleotide pools (Mohlmann et al., 2010), which availability in early stages of seedling development is critical for the success of germination (Stasolla et al., 2003). Nucleotides metabolism during germination have been studied mostly using radiolabeled metabolites or enzymatic assays with crude extracts (Ashihara, 1977; Guranowski and Pawełkiewicz, 1978; Guranowski and Barankiewicz, 1979; Ashihara, 1983; Florchinger et al., 2006) being the number of studies at molecular level scarce, and mainly restricted to the model plant *A. thaliana* (Jung et al., 2009; Cornelius et al., 2011). However, and despite the importance of purine catabolism in ureidic legumes, to our knowledge, this is the first study reporting a complete analysis of the regulation of nucleosides degradation during germination and early postgerminative development in legumes.

Our qRT-PCR, activity and immunodetection assays reveal a marked induction of NSHs expression and activity around the radicle emergence in both cotyledons and embryonic axes (Figures 3, 4), pointing to an important role of nucleosidases during germination and early seedling development. These results support earlier observations using radiolabeled purines and pyrimidines. Thus, in cotyledons of the ureidic legume *Phaseolus mungo*, most of the purine derivatives are salvaged for nucleotide and nucleic acid synthesis between the imbibition and the initiation of the germination, whereas degradation prevails in the later stage (Ashihara, 1983). Accordingly, in lupin cotyledons, increased activity of adenosine nucleosidase was observed from day 3 of germination, coincident with the reduction of the activities of recycling enzymes adenine and hypoxanthine-guanosine phosphoribosyltransferase and adenosine kinase (Guranowski and Pawełkiewicz, 1978; Guranowski and Barankiewicz, 1979), suggesting that the induction of NSHs marks a transition from salvage to degradation of purines. Likewise, in embryonic axes, high salvage was detected during early germination, while degradation and incorporation of radiolabeled ^14^C purines to ureides was prominent mainly in the later stages. Supporting these results, we observed an increase in ureides in embryonic axes at 3 DAI (Figure 6), concomitant with the increase in nucleosidase activity in both cotyledons and radicles. Regarding pyrimidines, several studies have shown that the pyrimidine nucleotide pools come mainly from salvage reaction at the first stage of germination to be replaced by *de novo* synthesis in the later stages (Ross and Murray, 1971; Ashihara, 1977; Stasolla et al., 2001), with nucleoside and nucleobase degradation all along the germinative process. Furthermore, evidence from *Arabidopsis NSH1* knockout and overexpression mutants indicate that nucleosidases play a crucial role in the regulation of the balance between uridine degradation and salvage, which, according to Jung et al. (2009), highlights the importance of a fine regulation of nucleosidase activity for the success of germination.

We have shown that two cytosolic nucleosidase genes are expressed during germination and seedling development and that expression of both genes are induced during seedling development. There were no remarkable differences between the expression pattern or the level of transcripts of *PvNSH1* and *PvNSH2* during germination and early postgerminative development, and we were unable to distinguish both proteins in our Western blot analyses (Figures 3, 4). However, when the crude extracts were assayed for nucleosidase activity using uridine as substrate, we found a significant reduction of nucleosidase activity compared to that with xanthosine, suggesting a prominent role of PvNSH2 during this developing stage and emphasizing the importance of purine catabolism in common bean during germination.

Once the seedling germinates, nucleosidase activity seems to be more important in radicles than in hypocotyls agreeing with previous results in *Arabidopsis* (Riegler et al., 2011). This may be related with the ability to catabolize nucleosides from the rhizosphere (Tokuhisa et al., 2010). In common bean cotyledons, high mobilization of compounds occurred about the 7th day of development (Lescano et al., 2016), which explains the elevated nucleosidase activity in senescent cotyledons (8 and 10 DAI). The discrepancy between the transcript level and the activity and the protein observed can be explained by the reduction in the total amount of protein in senescent cotyledons due to mobilization, which may affect the relative abundance of NSHs.

The induction of nucleosidase activity and the relative levels between radicles and cotyledons are coincident with the total nuclease activity assayed *in vitro* (Figure 5). In the late stages of germination, the demand of nucleotides and synthesis of nucleic acids decrease in cotyledons, increasing their degradation and export to the developing organs (Ashihara, 1983). In castor bean, degradation of nucleic acids and nucleotides, and transport of nucleosides and nucleobases from the senescent endosperm to the developing organs were also found (Kombrink and Beevers, 1983). This is consistent with the induction of total ssDNAse and NSHs activities observed in common bean cotyledons, and with previous reports showing the induction of two S1/P1 nucleases in senescent cotyledons (Lambert et al., 2016). It is not clear whether the nucleotides degraded in the cotyledons are transported to the growing tissue as nucleosides, nucleobases, or ureides, but the induction of NSH activity in cotyledons suggests that at least a part of them may be transported as bases and/or ureides. The precursor of ureides, xanthosine, and xanthine cannot be salvaged (Ashihara et al., 2020a), and we found a decrease of their pools in cotyledons just before radicle emergence (Table 3), suggesting ureide synthesis in cotyledons at early stages of germination. In addition, active synthesis of ureides at early and late germination in *P. mungo* cotyledons, as well as the inability to complete the degradation of ureides in common bean cotyledons has been reported (Ashihara, 1983; Quiles et al., 2009), which supports that these molecules should be transported to the axes for their degradation. By contrast, *P. mungo* seeds incubated with radiolabeled adenine in the presence of allopurinol, a xanthine dehydrogenase inhibitor, incorporate radioactivity into xanthine in the embryonic axes (Ashihara, 1983; Quiles et al., 2009), and ureides decrease when excised axes are incubated with allopurinol (Ashihara, 1983; Quiles et al., 2009), indicating that atleast a fraction of the purines catabolized in the cotyledons are translocated to the axes as nucleobases and degraded to ureides within the axes.

In embryonic axes of common bean, a sharp increase in ssDNAse activity occurs when the radicle emerges in parallel to the increase in nucleosidase activity, ureides, and some nucleosides. An S1/P1 nuclease with a proposed role in development and two nucleotidases are also induced after the radicle protrusion (Lambert et al., 2014; Cabello-Diaz et al., 2015; Galvez-Valdivieso et al., 2020). Therefore, the coordinated expression of nucleases, nucleotidases, and nucleosidases in developing axes suggests that the nucleic acids stored during seed formation could serve as a potential reserve of nitrogen and phosphorus. This could be particularly relevant for species with large genomes or with high DNA content in seeds as result of endoreduplication. Interestingly, cotyledons and embryonic axes of *P. vulgaris* seeds contain endopolyploid nuclei (Rewers and Sliwinska, 2014).

**Figure 7:**
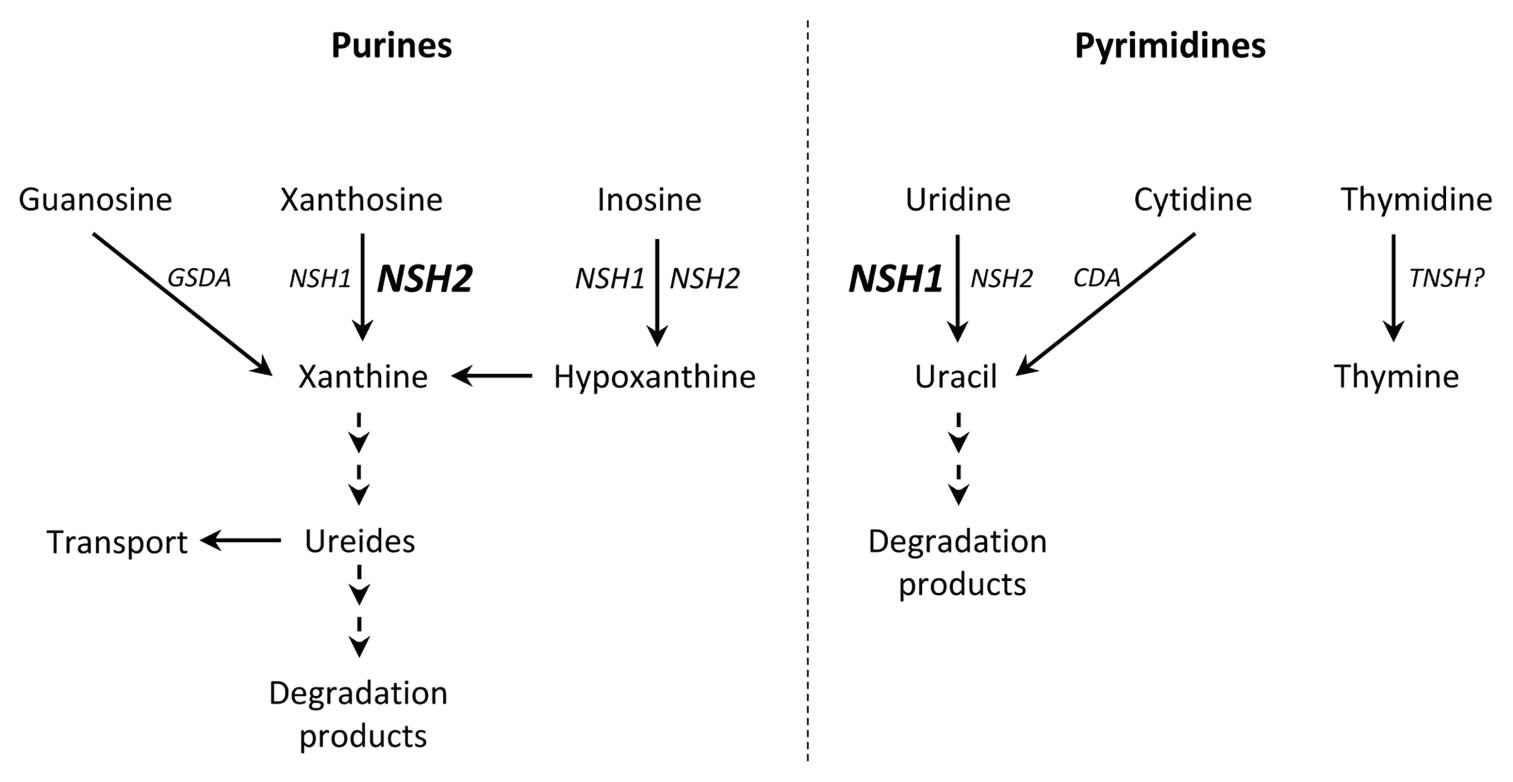
Hypothetical model of purine and pyrimidine degradation during common bean germination highlighting the role of PvNSH1 and PvNSH2. Nucleosidases are induced around radicle emergency. Bold and font size indicated a prominent role of this isoform. (GSDA, guanosine deaminase; CDA, cytidine deaminase; NHS1, PvNSH1; NSH2, PvNSH2; and TNSH, putative thymidine nucleosidase).

To summarize, the two cytosolic nucleosidases found in *P. vulgaris* present differential activities and substrate affinities, and seem to be functional without interacting with each other. Both *PvNSH1* and *PvNSH2*, and the nucleosidase activity boost around the radicle emergency, with no remarkable differences between them, indicating that both nucleosidases play an important role in the mobilization of nucleotides during germination and early postgerminative development. A scheme highlighting the putative role of PvNSH1 and PvNSH2 in this process is shown in Figure 7. According to this, PvNSH1 could play a prominent role in pyrimidine catabolism whereas PvNSH2 could be more involved in purine catabolism, to which also contributes PvNSH1. This prominent role of PvNSH2 in purine catabolism during common bean seedling development could be related to the importance of purine catabolism in ureidic legumes.

## Data Availability Statement

The original contributions presented in the study are included in the article/Supplementary Material, further inquiries can be directed to the corresponding author.

## Author Contributions

ED-G performed most of the experiments, analyzed the data, and reviewed the manuscript. GG-B performed and analyzed the LC-MS/MS experiment and reviewed the manuscript. IMG-M optimized the LC-MS method and performed the analyses. PP and MP acquired the funding and reviewed the manuscript. PP analyzed the data and conceived the project. GG-V designed the experiment, conceived and supervised the project, analyzed the data, and wrote the manuscript. All authors contributed to the article and approved the submitted version.

### Conflict of Interest

The authors declare that the research was conducted in the absence of any commercial or financial relationships that could be construed as a potential conflict of interest.

## Footnotes

^1^http://phytozome.jgi.doe.gov/pz/portal.html
