# Supplementary material for: Nucleoside Metabolism Is Induced in Common Bean During Early Seedling Development
Source: Front Plant Sci. 2021 Mar 25;12:651015. doi: 10.3389/fpls.2021.651015 (PMC8027947; doi:10.3389/fpls.2021.651015)
Supplement: Supplementary file 1 [file Data_Sheet_1.pdf]

Supplementary Table S1. Primers used in this work. Sequences read from 5' to 3'.

Purpose	Gene	Name	Sequence (5'-3')
Cloning	PvNSH1	clo-NSH1-F	GTATGCTCCCTCCAGTATCTA
		clo-NSH1-R	CCATGGTGAATTCTAATATCC
	PvNSH2	clo-NSH2-F	CTGATCTGCTTCTCAAACAAG
		clo-NSH2-R	TAGATAGAGAGGAGTTGGCACT
pET-30b+ cloning	PvNSH1	pET30-NSH1-SalI	GATGTCGACATGGCCTCGCAGGTGAACAGC
		pET30-NSH1-NotI	GGCGGCCGCTCGGGTTTCATCACCAGCTC
	PvNSH2	pET30-NSH2-SalI	AGTTCTGGTCGACTGGCAGCCGATACC
		pET30-NSH2-NotI	GCGGCCGCTGAAGAGTCCAAAAGG
pXCS-HAStrep cloning	PvNSH2	pXCS-NSH2-Hind	CTCAAGCTTATGGCAGCCGATACC
		pXCS-NSH2-SmaI	CCCGGGAGAGTCCAAAAGGCGAT
qRT-PCR	Actin-2	Act-2-F3	GCAATTCAGGCTGTCTTGTCTTTGT
		Act-2-R3	TAAATCACGGCCAGCAAGATCC
	UBQ	qUBQ-F3	TACATGCGATCTTGGACTGGC
		qUBQ-R3	GGGGCTTTTCTGGGTAGTCT
	PvNSH1	qNSH1-F5	TTGGCAATGCAATGACGGAAG
		qNSH1-R5	GGCGTTCCACCCTTCAATGG
	PvNSH2	qNSH2-F7	TGGCAACCAAAATTTTCCTCCC
		qNSH2-R7	GTGACTTTCCCAGGATTAAGTTTTGC

Supplementary Table S2. Percentage of similarity of PvNSH1 and PvNSH2 with other NSHs from plants.

	**PvNSH1**	**PvNSH2**
PvNSH1	-	51.6
PvNSH2	51.6	-
AtNHS1	75.1	50.3
AtNSH2	53.0	77.6
C. arietinum NSH1	82.0	49.7
C. arietinum NSH2	50.8	87.0
G. max NSH1.1	87.3	56.4
G. max NSH1.2	87.9	52.3
G. max NSH2.1	51.2	90.7
G. max NSH2.2	43.3	78.1
L. japonicus NSH1	82.3	51.9
L. japonicus NSH2	53.3	87.9
M. truncatula NSH1	82.6	50.9
M. truncatula NSH2.1	48.3	80.7
M. truncatula NSH2.2	51.1	87.6
N. tabacum NSH1	74.3	50.6
N. tabacum NSH2	50.3	81.6
O. sativa NSH1.1	71.6	49.4
O. sativa NSH1.2	68.5	48.4
O. sativa NSH2	53.4	72.3
P. trichocarpa NSH1	77.2	51.1
P. trichocarpa NSH2	52.2	83.2
S. lycopersicum NSH1	75.6	49.7
S. lycopersicum NSH2	51.2	82.2
T. aestivum NSH1.1	69.8	47.8
T. aestivum NSH1.2	68.3	52.2
T. aestivum NSH2	51.2	72.4
V. unguiculata NSH1	91.6	51.6
V. unguiculata NSH2	52.8	94.1
ZmNRH1a	49.7	70.6
ZmNRH1b	50.2	69.3
ZmNRH2a	71.1	50.9
ZmNRH2b	72.6	50.9
ZmNRH3	72.8	52.2

Supplementary Table S3. MS detection methods and limit of quantification (LOQ) of the investigated compounds.

a) Optimized mass spectrometric parameters and retention time (R_t) for target compounds

Metabolite	Formula	Theoretical mass (g mol^{-1})	Precursor ion (m/z)	Product ions (m/z)	DP (V)	CE (V)	R_t (min)
Xanthosine	$C_{10}H_{12}N_4O_6$	284.228	285.1	153.1<br>133.2	21	15<br>15	5.75
Inosine	$C_{10}H_{12}N_4O_5$	268.226	269.1	137.1<br>110.1	51	25<br>55	5.34
Guanosine	$C_{10}H_{13}N_5O_5$	283.241	284.1	152.1<br>110.1	76	23<br>57	5.43
Adenosine	$C_{10}H_{13}N_5O_4$	267.241	268.1	136.1<br>92	96	23<br>73	5.69
Uridine	$C_9H_{12}N_2O_6$	244.201	245	112.9<br>70	66	19<br>47	3.16
Thymidine	$C_{10}H_{14}N_2O_5$	242.229	243.1	127.1<br>117.1	56	17<br>17	5.88
Cytidine	$C_9H_{13}N_3O_5$	243.220	244.1	112.1<br>95	41	27<br>59	1.57
Guanine	$C_5H_5N_5O$	151.126	152	135<br>110	56	25<br>27	2.08
Uracil	$C_4H_4N_2O_2$	112.087	113	70<br>96.1	106	21<br>23	1.9
Cytosine	$C_4H_5N_3O$	111.102	112	95.1<br>52	76	23<br>41	1.17
Xanthine	$C_5H_4N_4O_2$	152.111	153	110.1<br>136.1	101	25<br>19	3.00
Hypoxanthine	$C_5H_4N_4O$	136.111	137	118.9<br>110	46	29<br>31	2.61
Adenine	$C_5H_5N_5$	135.127	136	109<br>119.1	111	33<br>29	1.88
Thymine	$C_5H_6N_2O_2$	126.113	127	109.8<br>56.1	66	21<br>29	3.90

b) Limit of quantification (LOQ) of the investigated compound.

Analytes	LOQ (ng mL^{-1})
Xanthosine	0.495
Inosine	0.890
Guanosine	1.253
Adenosine	1.987
Uridine	0.817
Thymidine	2.197
Cytidine	0.203

Analytes	LOQ (ng mL^{-1})
Guanine	0.353
Uracil	1.500
Cytosine	0.123
Xanthine	0.780
Hypoxanthine	0.517
Adenine	0.367
Thymine	1.063

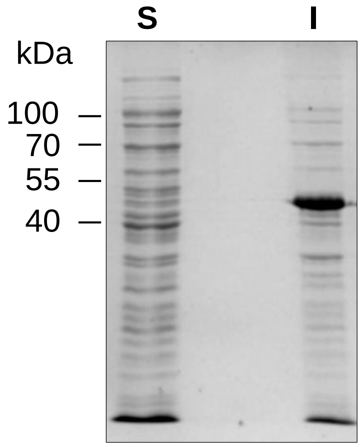

Supplementary Figure S1. Overexpression of PvNSH2 in *E. coli*. Coomassie stained gel. S: Soluble fraction; I: insoluble fraction.

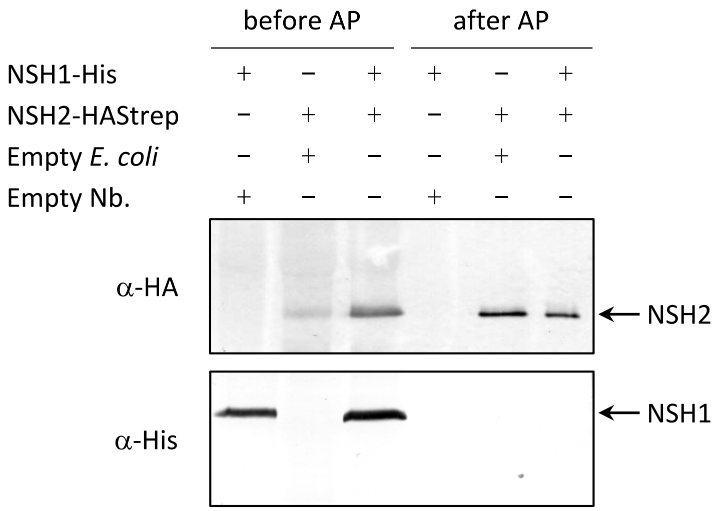

Supplementary Figure S2. Interaction of NSH1 and NSH2. Extracts of *E. coli* BL21 (DE3) and *N. benthamiana* overexpressing NSH1-His and NSH2-HAStrep, respectively were mixed for 12 h at 4 ºC. Western blot using anti-HA (upper panel) or anti-His (lower panel) was performed before and after affinity purification (AP) using Strep Tactin sepharose. Nb: *Nicotiana benthamiana.*

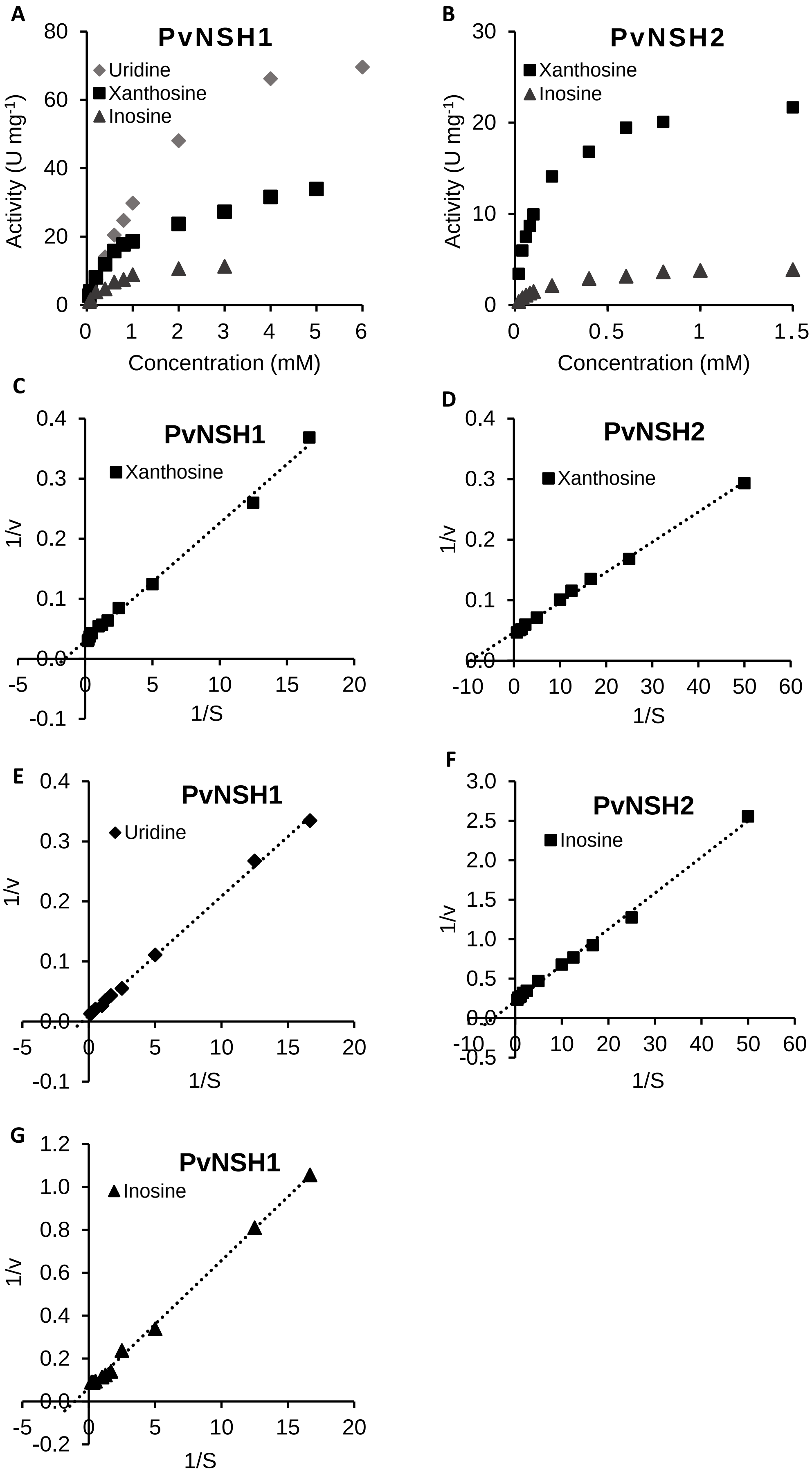

Supplementary Figure S3. Michaelis-Menten curves of PvNSH1 activity with xanthosine, uridine and inosine (A) and PvNSH2 with xanthosine and inosine (B). Lineweaver-Burk plot for PvNSH1 (C, E, G) and PvNSH2 (D, F).
